# Species composition and population dynamics of malaria vectors in three previously ignored aquatic systems in Sri Lanka

**DOI:** 10.1186/s12936-016-1316-4

**Published:** 2016-05-10

**Authors:** Achini W. Fernando, Sevvandi Jayakody, Hiranya K. Wijenayake, Gawrie N. L. Galappaththy, Mangala Yatawara, Jeevanie Harishchandra

**Affiliations:** Department of Aquaculture and Fisheries, Wayamba University of Sri Lanka, Makandura, Gonawila, Sri Lanka; World Health Organization, Yangon, Myanmar; Department of Zoology and Environmental Management, University of Kelaniya, Kelaniya, Sri Lanka; Anti Malaria Campaign, Colombo 05, Sri Lanka

**Keywords:** Agro well, Anopheline larvae, Clay quarry pit, Malaria, Granite quarry pit

## Abstract

**Background:**

In 2015 alone there were an estimated 214 million new cases of malaria across the globe and 438,000 deaths were reported. Although indigenous malaria has not been reported in Sri Lanka since 2012, to date 247 imported cases of malaria have been identified. Knowledge of the locations, behaviour and vectorial capacity of potential malarial vectors is therefore needed to prevent future outbreaks. Attention is now being focused on some previously ignored habitats.

**Methods:**

Active and abandoned granite and clay quarry pits, located in wet and intermediate zones, and agro wells located in the dry zone of Sri Lanka were mapped and sampled for 1 year, as potential mosquito breeding sites. Species composition and spatio-temporal variation in both malarial and other mosquito larvae were recorded.

**Results:**

A total of 18 species of mosquito larvae were identified. Other than *Anopheles culicifacies*, the primary malaria vector, five species of potential malaria vectors (*Anopheles vagus*, *Anopheles varuna*, *Anopheles nigerrimus*, *Anopheles peditaeniatus* and *Anopheles barbirostris*) were found in all three aquatic systems. Additionally, *Anopheles annularis* was found in granite quarries and *Anopheles subpictus* and *Anopheles pallidus* in both types of quarry, but only during the initial sampling. Apart from potential malaria vectors, mosquito larvae such as *Anopheles jamesii*, *Culex tritaeniorhynchus*, *Culex infula* and *Culex malayi* were found in all three habitats at least once during the sampling period. Apart from potential malaria vectors and other mosquito larvae common to all three aquatic systems, *Culex gelidus*, *Culex mimulus* and *Culex pseudo vishnui* were detected in agro wells. *Culex gelidus* was also detected in granite quarry pits. *Culex mimulus*, *Culex lutzia* and *Culex fuscocephala* were detected in clay quarry pits. Accordingly, a total of 14, 13 and 15 mosquito species were identified in agro wells, granite and clay quarry pits, respectively.

**Conclusions:**

Although zero occurrence of indigenous malaria has been achieved in Sri Lanka, the current study emphasizes the potential for future epidemics. The presence of native flora and fauna in abandoned granite and clay quarry pits and the need to extract drinking water from agro wells demand bio-sensitive control methods in these three aquatic systems.

## Background

Globally, malaria has caused catastrophic and formidable health problems and about 3.2 billion people remain at risk [1]. At present, approximately 80 % of malaria deaths are concentrated in just 15 countries, the majority of which are in Africa [1]. In Southeast Asia malaria is still prevalent in ten countries, with India, Indonesia and Myanmar accounting for 96 % of cases [1]. The major *Plasmodium* species in this region are *Plasmodium falciparum* and *Plasmodium vivax*. However, the vector system in Southeast Asia is complex and difficult to distinguish morphologically, hence non-vectors have often been mistakenly included in potential malaria vector checklists [2].

In Sri Lanka, the traditional malaria-endemic zone extends across three-quarters of the country, encompassing most of the dry zone and intermediate zones [3]. About ten major epidemics have occurred there, while the epidemic between 1934 and 1935 was the most serious [4]. The Anti-Malaria Campaign (AMC) was established in 1911 and, since its inception, has made several attempts to eradicate indigenous malaria and to prevent transmission within the country [4]. Even though no indigenous malaria cases were recorded in 2013 [3] and 2014 [1], there is a potential for epidemics to occur [5], as imported malaria cases are still being recorded [6] and regulations for chemoprophylaxis and screening on re-entry to the country are not strictly adhered to [5].

Globally, malaria is declining [1, 7]. However, more effort is being made to understand the role of previously overlooked or ignored habitats in sustaining mosquito populations, as it has emerged that despite efforts to eliminate mosquitoes from known habitats, other habitats are harbouring populations which could cause malaria [8]. Additionally, new aquatic systems are constantly being created due to human activities, such as mining and agriculture. But these systems have not been given due attention despite their proximity to human dwellings and their increase in numbers. The current study was conducted in three previously ignored aquatic systems in Sri Lanka. Selection was based on proximity to human dwellings, relative abundance and presence of water during most months of the year, together with a lack of previous anopheline vector data. Accordingly, agro wells and granite and clay quarry pits were selected. Agro wells are abundant in the dry zone (mean annual rainfall ≥1750 mm). They are intensively used by farmers for both agricultural and domestic purposes and are an integral part of any farmland in the dry zone of Sri Lanka. The diameter and depth of these wells can vary, depending on the location and depth of the ground water table. They are wide-mouthed and shallow. Granite and clay quarry pits are found in the wet (mean annual rainfall ≤2500 mm), intermediate (mean annual rainfall = 1750–2500 mm) and dry zones. They are present as both active and abandoned pits. The abandoned pits have become semi-naturalized, lentic water bodies and they now harbour a diversity of native flora and fauna. They are dynamic systems, with some of the abandoned quarries becoming active from time to time. This alters their connections, dimensions and resident aquatic organisms.

## Methods

### Estimating the larval density of quarry pits

Granite and clay quarry pits were selected in the wet and intermediate zones in the Ma oya River basin, which is the main production zone for granite and bricks in Sri Lanka. Initially all granite and clay quarry pits were mapped, using information extracted from Google Earth^®^, local people and government records. Between June and September 2011, the presence of mosquito larvae in these quarries was monitored using a 350-ml standard dipper, as described in ‘Guidelines to searching for mosquito breeding habitats: stagnant water and conducting larval survey’ [9].

**Fig. 1:**
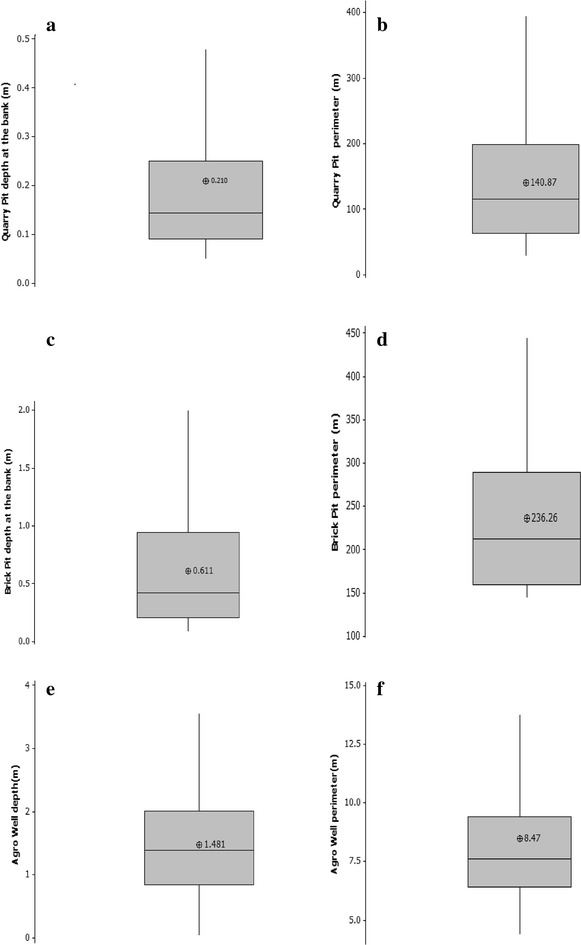
Box plots depicting mean depth and perimeter of granite quarry pits **a**, **b**, clay quarry pits **c**, **d** and agro wells **e**, **f** during the initial survey

From the initial survey, 41 clay quarry pits and 38 granite quarry pits were identified and their locations fixed using a hand-held GPS (GPS GAMIN-GPSMap60cs). Basic information about these mapped pits is given in Fig. 1a–d. No mosquitoes were detected in 38 and 24 % of clay and granite quarry pits, respectively. Of the 41 clay quarry pits, 38 were abandoned and of the 38 granite quarry pits, 33 were abandoned. All abandoned quarries appeared to be naturalized by native flora and fauna. From the abandoned quarries, ten with evidence of mosquito larvae presence were randomly selected for continuous monitoring of anopheline vectors and other mosquitoes, from February 2012 to June 2013.

In order to measure the larval density, a dipper was lowered gently at an angle of 45° to just below the surface, to ensure an undisturbed and uninterrupted flow of water, and 350-ml was collected with any larvae that might be present. Six dips per sampling position were made and the samples were pooled. Sampling positions were always 10 m apart and the number of sampling positions varied according to the size of the quarry. The depth of each sampling position was recorded, to the nearest cm, using the graduated handle of the dipper. The prevailing weather conditions (cloud cover, wind direction, rain), water turbidity and water temperature were also recorded for each quarry sampled. Sampling was always carried out between 08:00 and 14:00.

### Estimating the larval density of agro wells

An area with a known history of malaria outbreaks was selected from North Central province. Accordingly, 132 agro wells were identified and marked and sampled for mosquitoes in Wagollakada and Rathmale. Basic information about these agro wells is given in Fig. 1e, f. No mosquitoes were found in 66 % of the wells. Of the wells with mosquitoes, 36 were randomly selected for continuous monitoring. Buckets were used to draw 2-l samples of water from four sides of each well, with minimal disturbance to the water and any mosquito larvae present.

Once the larvae had been collected, they were transferred to labelled vials. In the laboratory, the species of third and fourth instar larvae were identified using standard guides [10]. The numbers of larvae per dip were then estimated and mean values for each granite and clay pit and for each month were calculated.

### Ethical committee

The Ethics Committee of Wayamba University of Sri Lanka in the Faculty of Livestock, Fisheries and Nutrition gave approval for this study, including the collection of mosquito larvae from aquatic systems for identification.

## Results

### Occurrence of difference anopheline and culicine larvae in granite and clay quarry pits and agro wells

A total of 18 species of mosquito larvae were identified in the current study. Other than *Anopheles culicifacies,* the primary malaria vector, five species of potential malaria vectors (*Anopheles vagus*, *Anopheles varuna*, *Anopheles nigerrimus*, *Anopheles peditaeniatus* and *Anopheles barbirostris*) were also identified in all three aquatic systems. Additionally, *Anopheles annularis* was found in granite quarries and *Anopheles subpictus* and *Anopheles pallidus* in both types of quarries, but only during the initial sampling.

Apart from potential malaria vectors, mosquito larvae such as *Anopheles jamesii*, *Culex tritaeniorhynchus*, *Culex infula* and *Culex malayi* were present in all three aquatic systems at least once during the sampling period.

**Fig. 2:**
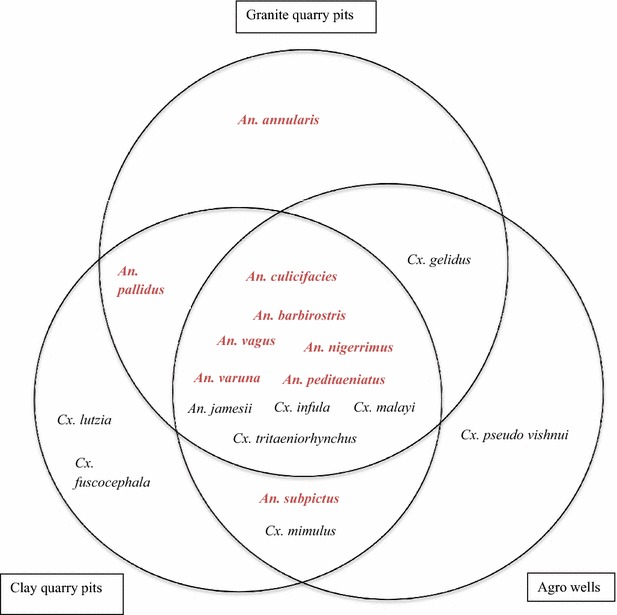
Species composition of mosquitoes recorded from granite quarry pits, clay quarry pits and agro wells. Potential malaria vectors are indicated in *bold red letters*

Besides the potential malaria vectors and other mosquito larvae common to all three aquatic systems, *Culex gelidus*, *Culex mimulus* and *Culex pseudo vishnui* were detected in agro wells. *Cx. gelidus* was also detected in granite quarry pits and *Cx. mimulus*, *Culex lutzia* and *Culex fuscocephala* were detected in clay quarry pits. Accordingly, a total of 13, 15 and 14 mosquito species were identified in granite and clay quarry pits and agro wells, respectively (Fig. 2).

### Fluctuation of larval density in granite quarry pits

**Fig. 3:**
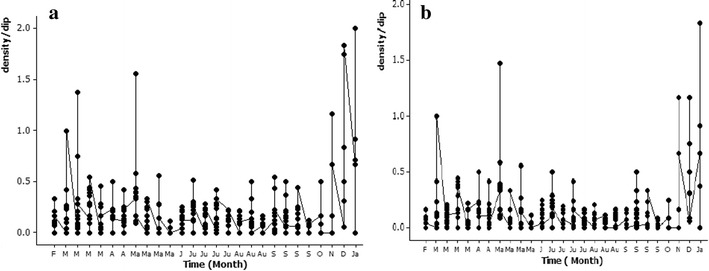
Fluctuation of larval density of **a** total anopheline and culicine, and **b** all potential malaria vector species from February 2012 to January 2013 in granite quarry pits

In granite quarry pits, at least one species of mosquito was detected at every sampling and a total of eight potential malaria vectors (*An. culicifacies*, *An. varuna*, *An. peditaeniatus*, *An. nigerrimus*, *An. vagus*, *An. barbirostris*, *An. pallidus*, *An. annularis*) were recorded (Table 1). The peak densities of all mosquitoes ($\bar{x}$ = 0.61 ± 0.72 SD per dip) and potential malaria vectors ($\bar{x}$ = 0.542 ± 0.675 SD per dip) were recorded in January 2013 (Fig. 3). Table 1 lists the species-specific population fluctuations for different months, showing that granite quarry pits served as a breeding site for at least one species of potential malaria vector throughout the study period (Fig. 3b).

**Table 1:** Summary of the larval density fluctuation from February 2012 to January 2013 in granite quarry pits

Species	Months of detection	Peak density ($\bar{x}$ ± SD per dip)
***An. culicifacies***	February 2012 to January 2013	January 2013 (0.13 ± 0.28)
***An. vagus***	March, April and August 2012	March 2012 (0.05 ± 0.09)
***An. varuna***	February to September 2012 and January 2013	September 2012 (0.09 ± 0.15)
***An. nigerrimus***	February to September 2012 and January 2013	January 2013 (0.07 ± 0.19)
***An. peditaeniatus***	February 2012 to January 2013	May 2012 (0.28 ± 0.30)
***An. barbirostris***	February to December 2012 and January 2013	December 2012 (0.47 ± 0.25)
*An. jamesii*	March 2012 to January 2013	March 2012 (0.07 ± 0.15)
*Cx. tritaeniorhynchus*	February, March, May, August, September, October 2012 and January 2013	December 2012 (0.12 ± 0.22)
*Cx. infula*	August and September 2012	September 2012 (0.02 ± 0.07)
*Cx. gelidus*	March, July and September 2012	March 2012 (0.08 ± 0.26)
*Cx. malayi*	December 2012	December 2012 (0.21 ± 0.57)
Potential malaria vector larvae	February 2012 to January 2013	January 2013 (0.54 ± 0.67)
Total anopheline and culicine mosquito larvae	February 2012 to January 2013	January 2013 (0.61 ± 0.72)

Potential malaria vectors are given in bold text

### Fluctuation of larval density in clay quarry pits

**Fig. 4:**
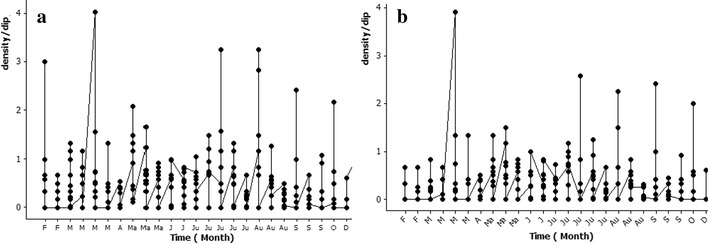
Fluctuation of larval density of **a** total anopheline and culicine, and **b** all potential malaria vector species from February 2012 to December 2012 in clay quarry pits

During the study period a total of 14 mosquito species, of which seven were potential malaria vectors (*An. culicifacies*, *An. vagus*, *An. varuna*, *An. nigerrimus*, *An. peditaeniatus*, *An. barbirostris*, *An. subpictus*), were recorded in clay quarry pits (Table 2). The peak densities of all mosquitoes ($\bar{x}$ = 0.87 ± 1.20 SD per dip) and potential malaria vectors ($\bar{x}$ = 0.724 ± 1.192 SD per dip) were recorded in March 2012 (Table 2). Clay quarry pits also served as breeding sites throughout the sampling period (Fig. 4).

**Table 2:** Summary of the larval density fluctuation from February 2012 to January 2013 in clay quarry pits

Species	Months of detection	Peak density ($\bar{x}$ ± SD per dip)
***An. culicifacies***	February, March, June, July and August 2012	March 2012 (0.37 ± 1.17)
***An. vagus***	March and September 2012	March 2012 (0.11 ± 0.31)
***An. varuna***	March to July 2012, August and September 2012, May 2013	August 2012 (0.23 ± 0.48)
***An. subpictus***	August and October 2012	August 2012 (0.28 ± 0.79)
***An. nigerrimus***	February to October 2012	August 2012 (0.19 ± 0.53)
***An. peditaeniatus***	February to October 2012	May 2012 (0.34 ± 0.37)
***An. barbirostris***	March to December 2012	September 2012 (0.30 ± 0.45)
*An. jamesii*	February to October 2012	May 2012 (0.40 ± 0.34)
*Cx. tritaeniorhynchus*	March, May, July, August, September and October 2012	August 2012 (0.22 ± 0.42)
*Cx. infula*	March 2012	March 2012 (0.06 ± 0.16)
*Cx. mimulus*	February 2012	February 2012 (0.06 ± 0.25)
*Cx. malayi*	February and December 2012	February 2012 (0.12 ± 0.50)
*Cx. lutzia*	March to June and August 2012	August 2012 (0.08 ± 0.18)
*Cx. fuscocephala*	March, May and June 2012	May 2012 (0.04 ± 0.11)
Potential malaria vector larvae	February to December 2012	March 2012 (0.72 ± 1.19)
Total anopheline and culicine mosquito larvae	February to December 2012	March 2012 (0.87 ± 1.20)

Potential malaria vectors are given in bold text

### Fluctuation of larval density in agro wells

**Fig. 5:**
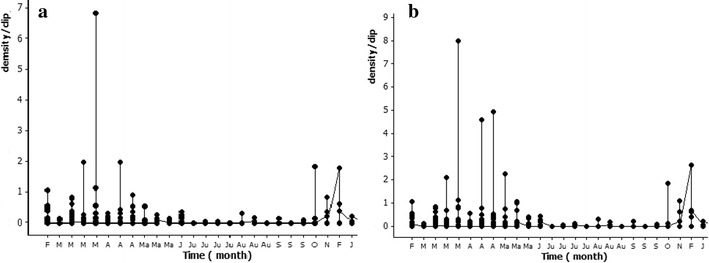
Fluctuation of larval density of **a** total anopheline and culicine, and **b** all potential malaria vector species from February 2012 to June 2013 in agro wells

A total of 14 mosquito species, of which seven were potential malaria vectors (*An. culicifacies*, *An. vagus*, *An. varuna*, *An. nigerrimus*, *An. peditaeniatus*, *An.subpictus*, *An. barbirostris*), were also recorded in agro wells (Table 3). The peak mosquito densities of all mosquitoes ($\bar{x}$ = 0.2 ± 0.58 SD per dip) (Fig. 5 a) and potential malaria vectors ($\bar{x}$ = 0.017 ± 0.40 SD per dip) were recorded in March 2012 (Fig. 5 b). The density of the primary malaria vector *An. culicifacies* peaked in October 2012 (0.02 ± 0.15 SD per dip).

**Table 3:** Summary of the larval density fluctuation from February 2012 to January 2013 in agro wells

Species	Months of detection	Peak density ($\bar{x}$ ± SD per dip)
***An. culicifacies***	March, April, May, June, August to November 2012 and February, June 2013	October 2012 (0.02 ± 0.15)
***An. vagus***	May, June, November 2012 and June 2013	June 2012 (0.000965 ± 0.01)
***An. subpictus***	April, May, June and July 2012	June 2012 (0.000965 ± 0.01)
***An. varuna***	February to June, September 2012 and February 2013	May, November 2012 (0.013 ± 0.05)
***An. nigerrimus***	February to May 2012 and February and June 2013	February 2013 (0.018 ± 0.12)
***An. barbirostris***	February to August, November 2012 and June 2013	March 2012 (0.062 ± 0.33)
***An. peditaeniatus***	May 2012, February and June 2013	February 2013 (0.0013 ± 0.017)
*An. jamesii*	March 2012 and June 2013	March 2012 (0.001 ± 0.01)
*Cx. tritaeniorhynchus*	February to May, August, September 2012, February and June 2013	May 2012 (0.029 ± 0.12)
*Cx. gelidus*	April 2012	April 2012 (0.001 ± 0.01)
*Cx. infula*	February to June 2012	March and May 2012 (0.0062 ± 0.04)
*Cx. mimulus*	March to June 2012	April 2012 (0.036 ± 0.25)
*Cx. malayi*	March to May 2012	May (0.009 ± 0.09)
*Cx. pseudo vishnui*	March 2012	March 2012 (0.00008 ± 0.001)
Potential malaria vector larvae	February 2012 to June 2013	March 2012 (0.017 ± 0.40)
Total anopheline and culicine mosquito larvae	February 2012 to June 2013	March 2012 (0.20 ± 0.58)

Potential malaria vectors are given in bold text

## Discussion

This study revealed the challenges in totally eradicating malaria, when existing aquatic systems are harbouring several species of potential malaria vectors. At a time when the Anti Malaria Campaign has declared Sri Lanka free from indigenous malaria [7], a thorough understanding of potential breeding sites is essential. The AMC achieved almost complete eradication of malaria from Sri Lanka in 1963, through a very effective integrated vector management programme, entomological surveys, indoor residual spraying, and prophylaxis [4]. However, between 1967 and 1968 the country faced another malaria epidemic [4, 11, 12]. Discontinuation of the vector management programme led to this resurgence of malaria, which is now considered to be a classical example of a post-eradication epidemic [12]. Although no indigenous malaria cases have been reported since 2012, a total of 95, 46 and 36 imported malaria cases were reported in 2013, 2014 and 2015, respectively [1, 5, 13]. Malaria is mainly imported by workers returning from Africa and other Southeast Asian countries [14], pilgrims returning from India [14, 15], legal and illegal emigrants from Africa [6], soldiers returning from foreign missions [15] and even multiday boat fishermen [13]. The three aquatic systems investigated in this study exist in close proximity to human dwellings, especially the agro wells. Accordingly, the probability of an epidemic cannot be ruled out.

There are other examples of malaria recurring after total eradication, such as in Mauritius, where malaria was reported in 1975 after total eradication in 1969 [16]. Therefore, any country that has achieved total eradication of indigenous malaria should still focus on factors that could lead to a recurrence of the disease. As long as mosquito vectors are present, with suitable climatic conditions and as long as malaria is still being imported, there is potential for further epidemics [13]. Notably, the results of the current study indicated that most of the potential malaria vectors that were found are present throughout the year in all three aquatic systems. Additionally, potential malaria vector species recorded in this study have also been recorded in marshlands [8], tanks [8, 17], streams [8, 18, 19], rice fields [8, 17], reservoirs [8, 20], seepage areas [17], irrigation canals [8, 17, 18, 21], agro wells [8], temporary water pools [8], brick fields [22], quarries [22], puddles [22], abandoned pits [23], animal foot prints [8], and wastewater and rainwater bodies [8]. Hence, the current study highlights the challenges to maintaining an indigenous malaria-free status. All three of the aquatic systems studied, as well as many of the habitats listed above, exist in close proximity to human dwellings, especially the agro wells. As an island nation, Sri Lanka’s capacity to minimize trans-boundary disease transmission is limited. Hence, the AMC should expect imported cases of malaria to continue in future. However, the AMC have several options for eradicating the resident vector populations, irrespective of the threat from neighbouring countries.

Of the three aquatic systems studied, agro wells are of primary concern, as they are present in the previous malaria belt of the country and are found within most properties. Their numbers are steadily increasing, although the number present in the North Central Province is not known. However, the published literature mentions that over 50,000 agro wells were constructed between 1980 and 1990 [24] Agro wells are used for obtaining drinking water in many households, which prevents the application of conventional chemical control methods. At present people also stock fish in agro wells, mainly *Aplocheilus parvus*, *Poecilia reticulata, Anabas testudineus, Oreochromis,* and *Channa* spp., as a means of biological control of mosquito larvae. It is therefore vital to continue studying the use of biological agents, especially the potential of native species [25]. Physical methods such as landfill and levelling, cleaning and water management [17] have limited use, as semi-naturalized quarries cannot be overly modified due to a requirement to maintain species diversity. Nevertheless, guidelines given at the point of granting excavation rights clearly indicate the need to refill pits after use, and if those guidelines are followed, any further abandonment of pits could be avoided.

At present, biological control of vectors and continuous monitoring of the three systems studied are recommended. The options for controlling mosquitoes in these aquatic systems include maintaining ecological integrity, so that natural predators control mosquito populations, the introduction of larvivorous fish [26, 27] or other natural predators [28–31] and testing of target-specific bio-pesticides [32, 33]. At the same time, the general public should be encouraged to use mosquito nets and medical practitioners should be provided with facilities for screening immigrants coming from destinations where malaria is suspected to exist.

## Conclusions

The presence of native flora and fauna in abandoned pits and the need to extract drinking water from agro wells demand alternative control methods in these three aquatic systems. Although eradication of indigenous malaria has been achieved in Sri Lanka, these previously unexplored habitats have potential for causing epidemics in the future, unless their malariogenetic potential is curtailed.
